# Percutaneous transforaminal full endoscopic decompression for the treatment of lumbar spinal stenosis

**DOI:** 10.1186/s12891-020-03566-x

**Published:** 2020-08-14

**Authors:** Peigen Xie, Feng Feng, Zihao Chen, Lei He, Bu Yang, Ruiqiang Chen, Wenbin Wu, Bin Liu, Jianwen Dong, Tao Shu, Liangming Zhang, Chien-Min Chen, Limin Rong

**Affiliations:** 1 Department of Spine Surgery, the 3rd Affiliated Hospital of Sun Yat-sen University, NO. 600 TianHe Road, TianHe District, GuangZhou, GuangDong Province China; 2 Minimally Invasive Spine Center, Changhua Christian Hospital, No.135, Nansiao St., Changhua city, Changhua county Taiwan

**Keywords:** Percutaneous transforaminal endoscopic surgery, Decompression, Lumbar spinal stenosis, Lumbar instability

## Abstract

**Background:**

One advantage of an endoscopic approach to treating lumbar spinal stenosis is preservation of spine stability and the adjacent anatomy, and there is a decrease in adjacent segment disc degeneration. The purpose of this study was to discuss the clinical efficacy of percutaneous transforaminal endoscopic decompression for the treatment of lumbar spinal stenosis (LSS).

**Methods:**

This is a retrospective study. From September 2012 to June 2017, 45 patients who were diagnosed with LSS underwent the treatment of percutaneous transforaminal endoscopic decompression (PTED) and were followed up at 1 week, 3 months and 1 year postoperatively. Low back pain and leg pain were measured by Visual Analogue Scale scoring methods (VAS-back and VAS-leg), while functional outcomes were assessed by using the Oswestry Disability Index (ODI). All patients had one-level lumbar spinal stenosis.

**Results:**

The most common type of stenosis was lateral recess stenosis (*n* = 22; 48.9%), followed by central stenosis (*n* = 13; 28.9%) and foraminal stenosis (*n* = 10: 22.2%). Regarding comparisons of VAS-back, VAS-leg, and ODI scores before and after operation, VAS and ODI scores significantly improved. The average leg VAS score improved from 7.01 ± 0.84 to 2.28 ± 1.43 (*P* < 0.001). The average ODI improved from 46.18 ± 10.11 to 14.40 ± 9.59 (P < 0.001). We also examined changes in ODI and VAS scores from baseline according to types of spinal stenosis, stenosis grade, spinal instability, and revision surgery in the same segment. The improvement percentage of leg VAS score was significantly less in patients with severe stenosis at both 3 months and 1 year postoperatively. The improvement percentages of ODI and leg VAS scores were significantly less in patients who had spinal instability and patients who had undergone revision surgery.

**Conclusion:**

The PTED approach seems to give good results for the treatment of LSS. However, this approach may be less effective for LSS patients who have lumbar instability or require revision surgery in the same segment.

## Introduction

Lumbar spinal stenosis (LSS) is a lumbar vertebrae disease resulting from degenerative changes caused by the narrowing of the central canal, the lateral recess, or neural foramen [1]. LSS is more commonly diagnosed in an aging population. Both surgical and conservative approaches have been used for the management of LSS. A surgical approach is recommended if well-conducted conservative management fails. It has been suggested that percutaneous endoscopic discectomy may be an efficient alternative to conventional open lumbar decompression surgery when treating lumbar spinal stenosis [2]. The argument for this is that open decompressions for spinal stenosis have a high complication rate and are painful to recover from but endoscopic techniques for decompressing herniated discs have shown low complication and morbidity rates.

It is a challenging task to initially manage degenerative spine disease in patients with advanced age and multiple comorbidities. Both percutaneous endoscopic discectomy and decompression have yielded optimal results and favorable long-term outcomes in patients over 70 years old who had various types of stenosis [3]. Percutaneous transforaminal endoscopic lumbar discectomy (PTED) is one of the most popular minimally invasive spine surgeries. It has been widely used for treating lumbar degenerative diseases. Although PTED is mainly conducted in elderly patients, the clinical efficacy of PTED in treating patients aged younger than 45 years who have lumbar disc herniation has been proved [4]. The endoscopic approach is considered as a safe and effective minimally invasive surgery [5, 6]. It is associated with better outcomes, small incisions, less damage to human tissues, lower complication rate, and shorter hospitalization times [7, 8]. Lumbar interbody fusion surgery has the advantages of a high fusion rate and an obvious decompression effect, but it causes great damage to the paravertebral muscles and facet joints. It has been reported that adjacent segment disc degeneration may occur due to increased mechanical stress on discs adjacent to the fusion. Besides, elderly patients with severe osteoporosis were prone to internal fixation failure after pedicle screw fixation [9]. One advantage of the endoscopic approach is the preservation of spine stability and the adjacent anatomy, and there is a decrease in adjacent segment disc degeneration. It has been argued whether decompression alone or decompression with concomitant fusion yields better results when treating LSS, and only a few studies investigated the curative effect of PTED for the treatment of LSS. Therefore, this study aimed to evaluate the clinical efficacy of PTED and to find out the prognostic factors of PTED for the treatment of LSS.

## Material and method

This is a retrospective study, and the Institutional Review Board of the Third Affiliated Hospital of Sun Yat-Sen University approved the study. The requirement for informed consent from each patient to use their clinical data for research purposes was waived. Patients were included if they had met all of the following inclusion criteria: 1) neurogenic intermittent claudication or radicular irritation with or without sensory loss; 2) unilateral radiating leg pain; 3) concordant imaging diagnosis of lumbar stenosis; 4) failure of conservative treatment for at least 3 months. The patients were excluded from this study if they had 1) potential mental illness; 2) a grade 2 lumbar spondylolisthesis; 3) multilevel lumbar spinal stenosis; or 4) severe scoliosis. From September 2012 to June 2017, a total of 45 patients with unilateral lumbar stenosis were consecutively enrolled. All of them met the inclusion criteria and were treated with percutaneous transforaminal endoscopic decompression.

### Imaging

**Fig. 1:**
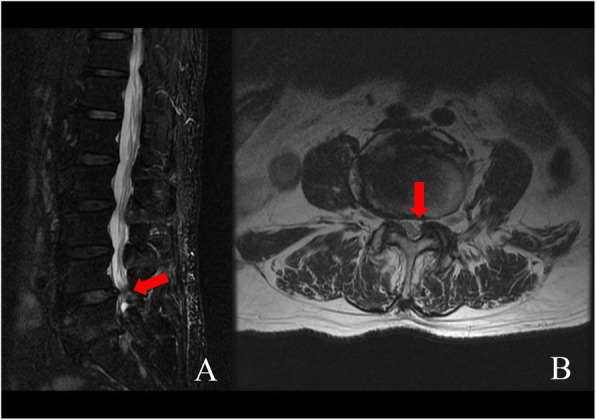
A patient diagnosed with L4/5 spinal stenosis on MRI; (**a**) Sagittal view demonstrates spinal stenosis at the L4/L5 level (**b**) Transverse views of spinal stenosis and lateral recess stenosis

**Fig. 2:**
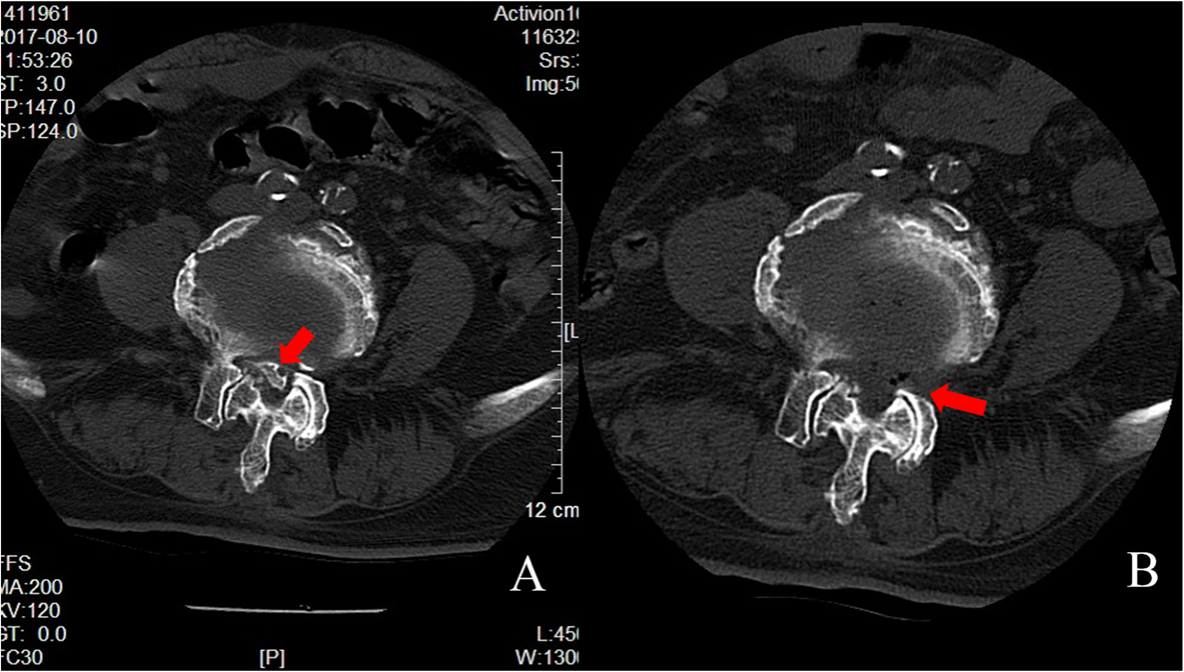
CT image showed (**a**) L4/L5 lumbar spinal stenosis and calcified lumbar disc herniation (**b**) Lumbar foraminoplasty (left) and decompression with removal of the calcified lumbar disc herniation (indicated by red arrows)

All patients were evaluated before the operation via computed tomography (CT) and magnetic resonance imaging (MRI). The severity of LSS was graded based on the observed morphology of the dural sac on image findings (Fig. 1) according to the method by Schizas [10]. We defined grade A as mild stenosis, B as moderate stenosis, C as severe stenosis. The CT images showed L4/L5 lumbar spinal stenosis and calcified lumbar disc herniation (Fig. 2). The locations of stenosis were classified as central canal, lateral recess, and foraminal narrowing of the spine. Dynamic X-ray images were used to examine spinal instability or backward slippage of the vertebral body.

### Surgical method (L4/L5)

Patients were placed in the prone position with the lumbar spine in mild flexion on a radiolucent table, and a C-arm fluoroscopy machine was used. The entry point of the needle was selected at a distance of 8–12 cm from the midline and was situated just above the facet joint on the lateral view. After local anesthesia, a puncture needle (18 gauge) was inserted by a posterolateral approach with a lateral view. After infiltrating 15–20 mL of 0.5% lidocaine in the intervertebral foramen, the needle was replaced with a 1-mm-diameter guidewire. Then a 22-gauge needle was inserted into the disc, followed by the injection of contrast medium (9 ml of iohexol with 1 ml of methylene blue) into the disc. A blunt tapered cannulated obturator was passed over the guide wire under fluoroscopic guidance. Sequential protective cannulas were introduced over the obturator until the final protective cannula was placed in the proper position. Foraminoplasty was performed using a 10-mm-diameter trephine via the transforaminal approach. After that, the protective cannula was replaced with an 8-mm working cannula. The endoscope (SPINENDOS GmbH, Munich, Germany) was positioned through a working casing pipe that was inserted through an 8-mm skin incision centered on a guidewire. The prominent intervertebral disc was stained using methylene blue and then removed by using a pituitary rongeur under endoscope. Next, the hypertrophied ligamentum flavum, facet joints, and anterior herniated disc were resected to achieve decompression. Epidural bleeding was controlled with a radiofrequency probe (SPINENDOS GmbH, Munich, Germany) under saline irrigation. Lastly, the working cannula and the endoscope were removed and the skin was sutured.

### Outcome measurement

Clinical outcomes were evaluated using the Visual Analog Scale (VAS; ranging from 0 to 10) to score pain [11] and the Oswestry Disability Index (ODI; ranging from 0% to 100% with higher scores meaning more severe disability) [12] preoperatively and postoperatively at 1 week, 3 months, and 1 year. The clinical variables include preoperative comorbidities, operation time, type of spinal stenosis, stenosis grade, spinal instability, and level of decompressed disc. Spinal instability was defined as greater than 3 mm translational motion and greater than 10 degrees angular motion (between L1 and L5) and greater than 4 mm translational motion and greater than 20 degrees angular motion (at L5/S1) [13].

### Statistical analysis

Data are expressed as the mean ± standard deviation for continuous variables. The Friedman test was performed to make comparisons of pain changes over time after percutaneous transforaminal endoscopic decompression. For spinal stenosis type and severity of stenosis, the improvement percentage of ODI and VAS scores were compared using the independent-samples Kruskal-Wallis test. The Mann-Whitney U test was used to make comparisons of improvement percentages of ODI and VAS scores based on spinal instability status and revision surgery status. A *P* value < 0.05 was considered to indicate statistical significance, and all tests were two-tailed. All statistical analyses were performed on a personal computer with the statistical package SPSS for Windows (Version 16.0, Chicago, IL, USA).

## Results

Demographic characteristics and clinical data of all patients are presented in Table 1. The patients consisted of 20 men and 25 women, ranging in age from 45 to 82 years (mean age 62 years). There was no statistically significant difference between the two groups with respect to age, BMI, spinal instability, the type of spinal stenosis, recurrent stenosis, and preoperative VAS and ODI scores. The rate of transient dysesthesia was 4.4% in the PTED group. Regarding the types of spinal stenosis, the most common type was lateral recess stenosis (*n* = 22), followed by central stenosis (*n* = 13) and foraminal stenosis (*n* = 10). Most patients had a moderate stenosis grade (*n* = 19). All patients had one-level (L3/4, *n* = 5; L4/5, *n* = 23; L5/S1, *n* = 17) lumbar spinal stenosis. Regarding comparisons of VAS-back, VAS-leg, and ODI scores before and after operation, VAS and ODI scores significantly improved. The preoperative VAS score for low back pain was 6.70 ± 1.15, while postoperative 1-week, 3-month, and 1-year VAS scores for low back pain were 3.07 ± 1.09, 2.90 ± 1.13 and 2.58 ± 1.11, respectively (P  <  0.001). The preoperative VAS-leg score was 7.01 ± 0.84, while VAS-leg scores for postoperative 1 week, 3 months and 1 year were 2.63 ± 1.23, 2.44 ± 1.33 and 2.28 ± 1.33, respectively (P  <  0.001). The preoperative ODI score was 46.18 ± 10.11, while postoperative ODI scores for 1 week, 3 months and 1 year were 19.29 ± 9.63, 16.31 ± 9.87 and 14.40 ± 9.59, respectively (P  <  0.001) (Table 2). Regarding types of spinal stenosis, there were no significant differences according to the improvements of ODI and VAS scores at 1 week, 3 months and 1 year. However, the improvement percentage of leg VAS score was significantly less in patients with severe stenosis at both 3 months and 1 year postoperatively (Table 3).

**Table 1:** Demographic characteristics and clinical data of 45 patients with lumbar spinal stenosis (LSS)

Characteristics		Men *n* = 20 (%)	Women *n* = 25 (%)	P	Total *N* = 45 (%)
Age (yrs)	mean ± S.D.	59.90 ± 9.60	62.80 ± 7.22	0.192	61.51 ± 8.38
BMI (kg/m^2^)	mean ± S.D.	22.70 ± 2.82	22.11 ± 2.94	0.385	22.37 ± 2.87
Spinal instability				1	
	Yes	4 (20.0)	6 (24.0)		10 (22.2)
	No	16 (80.0)	19 (76.0)		35 (77.8)
Revision surgery					
	Yes	4 (20.0)	4 (16.0)	1	8 (17.8)
	No	16 (80.0)	21 (84.0)		37 (82.2)
Type of spinal stenosis				0.104	
	Foraminal stenosis	2 (10.0)	8 (32.0)		10 (22.2)
	Lateral recess stenosis	13 (65.0)	9 (36.0)		22 (48.9)
	Central stenosis	5 (25.0)	8 (32.0)		13 (28.9)
Stenosis grade				0.611	
	Mild	5 (25.0)	9 (36.0)		14 (31.1)
	Moderate	10 (50.0)	9 (36.0)		19 (42.2)
	Severe	5 (25.0)	7 (28.0)		12 (26.7)
Coexisting disease					
	LSS alone	8 (40.0)	12 (48.0)	0.764	20 (44.4)
	Hypertension	5 (25.0)	10 (40.0)	0.352	15 (33.3)
	Diabetes	5 (25.0)	2 (8.0)	0.214	7 (15.6)
	Rheumatoid arthritis	0 (0)	2 (8.0)	0.495	2 (4.4)
	Coronary heart disease	4 (20.0)	4 (16.0)	1	8 (17.8)
	Osteoarthritis	4 (20.0)	1 (4.0)	0.155	5 (11.1)
Level of decompressed disc				0.017	
	L3–4	4 (20.0)	0 (0)		4 (8.9)
	L4–5	6 (30.0)	16 (64.0)		22 (48.9)
	L5-S1	10 (50.0)	9 (36.0)		19 (42.2)
Complication	Transient dysesthesia	1 (5.0)	1 (4.0)	1	2 (4.4)
Recurrent stenosis				0.383	
	Yes	4 (20.0)	2(8.0)		6(13.3)
	No	16(80.0)	23(92.0)		39(86.7)
Preoperative					
VAS-back	mean ± S.D.	6.92 ± 1.30	6.52 ± 1.00	0.153	6.7 ± 1.15
VAS-leg	mean ± S.D.	7.18 ± 0.82	6.88 ± 0.85	0.265	7.01 ± 0.84
ODI scores (%)	mean ± S.D.	47.70 ± 10.31	44.96 ± 9.99	0.403	46.18 ± 10.17
Postoperative 1 yr					
VAS-back	mean ± S.D.	2.72 ± 1.15	2.46 ± 1.08	0.464	2.58 ± 1.11
VAS-leg	mean ± S.D.	2.60 ± 1.50	2.02 ± 1.35	0.243	2.28 ± 1.43
ODI scores (%)	mean ± S.D.	14.80 ± 9.48	14.08 ± 9.86	0.764	14.40 ± 9.59

*BMI* body mass index, *VAS* visual analog scale, *ODI* Oswestry disability index, Values expressed as the mean ± S.D

**Table 2:** Comparisons of VAS and ODI scores before and after operation in patients with LSS

Variables	Preoperative Mean ± S.D.	Postoperative 1 week Mean ± S.D.	Postoperative 3 months Mean ± S.D.	Postoperative 1 year Mean ± S.D.	P
VAS for low back pain	6.70 ± 1.15	3.07 ± 1.09	2.90 ± 1.13	2.58 ± 1.11	< 0.001
VAS for leg pain	7.01 ± 0.84	2.63 ± 1.23	2.44 ± 1.33	2.28 ± 1.43	< 0.001
ODI	46.18 ± 10.11	19.29 ± 9.63	16.31 ± 9.87	14.40 ± 9.59	< 0.001

*VAS* visual analog scale, *ODI* Oswestry disability index

**Table 3:** Changes in ODI and VAS scores from baseline according to the type of spinal stenosis and stenosis grade

		Type of spinal stenosis		
Outcome	Foraminal stenosis	Lateral recess stenosis	Central stenosis	P
ODI (%)				
1wk	53.73 ± 21.65	58.46 ± 20.23	59.64 ± 15.28	0.730
3mo	59.09 ± 19.48	66.17 ± 21.20	65.45 ± 17.37	0.320
1 yr	65.03 ± 21.19	69.32 ± 21.12	69.76 ± 16.65	0.672
VAS for low back pain (%)				
1wk	51.76 ± 12.38	55.95 ± 12.15	55.87 ± 9.77	0.723
3mo	54.35 ± 8.06	57.87 ± 13.67	59.46 ± 13.72	0.696
1 yr	57.95 ± 8.77	64.45 ± 14.93	62.83 ± 12.84	0.537
VAS for leg pain (%)				
1wk	62.10 ± 19.66	62.73 ± 16.72	61.74 ± 17.53	0.980
3mo	71.57 ± 22.16	64.55 ± 16.86	61.14 ± 19.59	0.351
1 yr	75.68 ± 20.68	66.67 ± 19.14	62.43 ± 22.31	0.172
		Stenosis grade		
Outcome	Mild	Moderate	Severe	P
ODI (%)				
1wk	61.69 ± 18.44	59.30 ± 18.70	50.71 ± 19.68	0.204
3mo	69.98 ± 18.10	67.75 ± 17.79	52.54 ± 20.34	0.132
1 yr	73.53 ± 17.33	73.16 ± 15.84	55.23 ± 22.55	0.069
VAS for low back pain (%)				
1wk	59.22 ± 12.25	55.43 ± 8.37	49.36 ± 13.19	0.088
3mo	60.92 ± 14.61	60.03 ± 10.32	49.68 ± 10.49	0.037
1 yr	67.16 ± 16.51	65.06 ± 8.81	53.16 ± 10.57	0.010
VAS for leg pain (%)				
1wk	64.14 ± 16.12	64.41 ± 18.22	56.83 ± 17.06	0.301
3mo	71.33 ± 17.15	68.00 ± 19.32	53.32 ± 15.69	0.044
1 yr	74.82 ± 15.90	71.42 ± 20.84	52.56 ± 18.36	0.018

*VAS* visual analog scale, *ODI* Oswestry disability index

In terms of changes in ODI and VAS scores from baseline, the improvement percentages of VAS-back and VAS-leg scores were significantly less in patients with spinal instability. Similar results were found in patients who previously had operations in the same segment. Patients who had undergone revision surgery in the same segment had significantly less improvement in ODI postoperatively (Table 4).

**Table 4:** Changes in ODI and VAS scores from baseline according to spinal instability and revision surgery

Outcome	Had spinal instability	No spinal instability	P
ODI (%)			
1wk	43.61 ± 14.51	61.79 ± 18.29	0.003
3mo	45.71 ± 12.61	69.73 ± 17.92	0.001
1 yr	48.89 ± 15.48	74.10 ± 16.97	< 0.001
VAS for low back pain (%)			
1wk	47.05 ± 6.88	57.26 ± 11.53	0.002
3mo	45.89 ± 8.76	60.88 ± 11.48	0.001
1 yr	52.40 ± 8.23	65.44 ± 12.96	0.003
VAS for leg pain (%)			
1wk	50.88 ± 15.43	65.57 ± 16.47	0.012
3mo	48.52 ± 16.03	69.87 ± 16.95	0.003
1 yr	50.62 ± 20.71	72.26 ± 18.01	0.006
Outcome	Had revision surgery	No revision surgery	P
ODI (%)			
1wk	46.57 ± 15.68	60.17 ± 18.93	0.018
3mo	46.95 ± 13.84	68.16 ± 18.66	0.006
1 yr	48.32 ± 18.56	72.86 ± 17.09	0.001
VAS for low back pain (%)			
1wk	47.03 ± 8.91	56.71 ± 11.29	0.009
3mo	46.96 ± 8.68	59.84 ± 12.14	0.007
1 yr	50.62 ± 9.11	65.12 ± 12.56	0.003
VAS for leg pain (%)			
1wk	52.19 ± 17.02	64.49 ± 16.68	0.048
3mo	49.53 ± 17.61	68.50 ± 17.55	0.018
1 yr	48.38 ± 21.03	71.57 ± 18.18	0.011

*VAS* visual analog scale, *ODI* Oswestry disability index

## Discussion

The results of this study demonstrate that percutaneous transforaminal endoscopic decompression can be an effective treatment for elderly patients with lumbar spondylosis and spinal stenosis. Improvements in ODI and VAS scores were observed. Lumbar stenosis is an aging-related degenerative spine disease. The conservative approach might be the first option in patients with LSS and advanced age. Epidural injection of glucocorticoids plus lidocaine is commonly used in the treatment of LSS. Recently, it has been reported glucocorticoids plus lidocaine offered no short-term or long-term benefits as compared with epidural injection of lidocaine alone in the treatment of LSS [14, 15]. In a combined as-treated analysis, patients who underwent surgery showed significantly more improvement in all primary outcomes than did patients who were treated non-surgically. The efficacy of surgery for spinal stenosis has been reported in a randomized cohort controlled trial [16]. The surgical complication rates of open laminectomy for patients with LSS were from 4.8 to 8.8% [17, 18]. Because of such high surgical complication rates, endoscopic techniques such as PTED are preferred by most surgeons when treating patients who have high risks of side effects from general anesthesia. Currently, successful clinical outcomes have been achieved in LSS patients treated with full-endoscopic decompression for the defined indications [19]. Endoscopic approaches have also been widely performed for elderly patients with soft disc herniation and spinal stenosis. In a case series study, 85 patients having lumbar lateral recess stenosis with or without combined herniated discs were treated with percutaneous lumbar foraminoplasty and percutaneous endoscopic lumbar discectomy (PLF-PELD) and 90.6% of them reported the outcome as satisfactory [20]. However, some difficulty with full-endoscopic operations have been reported, such as incomplete removal of disc fragments, a steep learning curve, and recurrence [21]. The rate of incomplete removal of a herniated disc was 2.8 (283/10,288) in patients treated with PELD [22]. The recurrence rates following full-endoscopic operation for a herniated disc were from 5 to 6.2% [23, 24]. Elderly patients (age ≥ 60 years) and patients with diabetes had a higher risk of surgical failure during a full-endoscopic operation [25]. A modified PLF-PELD technique for patients with complex uncontained lumbar herniated discs has been reported, and 3.7% (5/134) of patients treated with it had recurrent herniation at the same level [26]. In the present study, we found that LSS patients treated with percutaneous transforaminal endoscopic decompression were satisfied with clinical outcomes regarding the improvement of ODI and VAS scores. However, a similar result was not found for LSS patients with lumbar instability. Fusion surgery is reserved for patients with coexisting instability; however, an increasing trend of fusion surgery between 2004 and 2009 was reported in a nationwide study [27]. The rate of fusions for LSS patients without degenerative instability increased from 13.5 to 21.4%, whereas the rate of decompressions alone decreased from 67.2 to 59.1%. Treatments of decompression with concomitant fusion for LSS patients have been reported. However, it has been argued whether decompression alone or decompression with concomitant fusion leads to better results for LSS patients [28]. The efficacy of decompression with concomitant fusion for LSS is unclear. Although minimally invasive lumbar interbody fusion surgery with percutaneous pedicle screw fixation, decompression of the spinal canal and intervertebral fusion under the dilatation channel relatively protect the paravertebral muscles and facet joints of the lumbar spine, adjacent segment disc degeneration problems remain [29]. A meta-analysis study revealed that additional fusion in the management of LSS yielded no clinical improvements over decompression alone [30]. A similar result was found in the management of degenerative LSS. Radiological investigations and patient-reported outcomes have been used to assess the efficacy of surgery for spinal disorders. Fusion has been associated with reduced disc space height at the adjacent segment and increased adjacent segment degeneration, but it had no influence on patient self-rated outcomes as well as VAS and ODI scores [31]. Due to inconsistent outcomes of reported studies, it is difficult to conclude an appropriate surgical approach for the treatment of LSS patients with spinal instability. Decompression alone cannot effectively improve the problem of subsidence of the intervertebral space and lumbar instability; therefore, LSS patients with lumbar instability tend to have poor postoperative results [6]. In the current study, we found that LSS patients with lumbar instability who underwent percutaneous transforaminal endoscopic decompression could immediately experience pain relief but had less improvement at 3 months postoperatively compared to LSS patients without lumbar instability. Similar results were found in those who had reoperations in the same segment as well.

### Limitations

The present study also has some limitations. Due to it being a retrospective study, a proper control group is lacking. Besides, all surgeries were performed by a single neurosurgeon and the improvement in surgical performance over time has not been evaluated. Also, the data were collected at a single medical center in China, which may somewhat limit the applicability of the study’s results. Larger scale studies are required to further verify the findings of the present study.

## Conclusions

The PTED approach seems to give good results for the treatment of LSS. However, this approach may be less effective for LSS patients who have lumbar instability or require revision surgery in the same segment.

## Data Availability

The datasets used and/or analysed during the current study are available from the corresponding author on reasonable request.

## Abbreviations

ODI: Oswestry disability index
VAS: Visual analog scale
CT: Computed tomography
MRI: Magnetic resonance imaging
LSS: lumbar spinal stenosis
